# Prevalence and determinants of mother and newborn skin-to-skin contact in The Gambia: a secondary data analysis

**DOI:** 10.1186/s42506-020-00050-1

**Published:** 2020-08-12

**Authors:** Michael Ekholuenetale, Adeyinka Onikan, Charity Ehimwenma Ekholuenetale

**Affiliations:** 1 Department of Epidemiology and Medical Statistics, Faculty of Public Health, College of Medicine, University of Ibadan, Ibadan, Nigeria; 2 Project Management Unit, Management Sciences for Health, Abuja, Nigeria; 3 Department of Economics, Benin Study Center, National Open University of Nigeria, Benin City, Nigeria

**Keywords:** Newborn, The Gambia, Skin-to-skin, Thermal care, Mother

## Abstract

**Background:**

Skin-to-skin contact (SSC) between mother and the newborn brings many benefits including its potential to promote the survival of the newborn. Nevertheless, it is a practice that is underutilized in many resource-constrained settings including The Gambia where a high rate of maternal and child mortality has been reported. In this study, we examined the prevalence and determinants of mother and newborn SSC in The Gambia.

**Methods:**

We used secondary data from The Gambia Multiple Indicator Cluster Survey (MICS)—2018. Data from 9205 women between 15-49 years who gave birth within 5 years of the survey was extracted for the analysis. Percentages and chi-square test were used for analyses. The significant variables from chi-square test were included in the multivariable binary logistic regression model to calculate the adjusted odds ratios (with corresponding 95% CI) of the factors associated with mother and newborn SSC.

**Results:**

The results of this study showed that the national prevalence of mother and newborn SSC was 35.7%. Across local government areas; Mansakonko (47.8%) and Kerewan (44.2%) had the highest prevalence, while Basse (28.5%) and Brikama (26.5%) had the least prevalence of mother and newborn SSC in The Gambia. Based on results from the logit model, normal weight (at least 2.5 kg) children were 1.37 times as likely to have mother and newborn SSC, compared with the low birthweight (< 2.5 kg) children (OR = 1.37; 95% CI: 1.05, 1.78). In addition, there was 38% increase in the odds of rural women who reported mother and newborn SSC, compared with urban women (OR = 1.38; 95% CI: 1.06, 1.79). Women who delivered at health facility were 3.35 times as likely to have mother and newborn SSC, compared with women who delivered at home (OR = 3.35; 95% CI: 2.37, 4.75). Furthermore, women who initiated antenatal care (ANC) after the first trimester had 21% reduction in the odds of mother and newborn SSC, compared with women who initiated ANC within the first trimester (OR = 0.79; 95% CI: 0.68, 0.93).

**Conclusion:**

The prevalence of mother and newborn SSC was low. In addition, geographical residence, birth weight, urban-rural residential status, place of delivery, and timing to ANC initiation were associated with mother and newborn SSC. There is a need to promote institutional based delivery using skilled birth attendance, promote early ANC initiation and healthy fetal growth.

## Introduction

Movement from the womb to the external world is a major event in the life of any individual. It is an event whereby the newborn adapts to the new world outside the mother’s womb and needs close contact between mother and the newborn to initiate an avenue for bonding and to promote the neurological and physiological development of the child together with, make the mother confident in her capacity to nurse her child [1]. Mother and newborn SSC after delivery involves placing the newborn on the mother’s chest for about 1 h. The healthy full-term newborn placed in SSC, would commence breastfeeding within 1 h of age [2]. The World Health Organization (WHO) has provided a standard definition of mother and newborn SSC: “when the infant is placed prone on the mother’s abdomen or chest in direct ventral-to-ventral skin-to-skin contact. Immediate skin-to-skin contact is done immediately after delivery, less than 10 minutes after birth. Early skin-to-skin contact was defined as beginning any time from delivery to 23 hours after birth. Skin-to-skin contact should be uninterrupted for at least 60 minutes” p.5 [3].

In spite of the efforts to improve children’s survival in sub-Saharan Africa (SSA), the death of a child within the first month of life has remained unabated [4]. Approximately 99% of all newborn deaths occur in resource-constrained settings, with about two-thirds occurring in Africa and Asia [5]. Improvements in neonatal survival rates are important for countries to meet the Sustainable Development Goals (SDGs) targets, which characterize global efforts toward improved health care-seeking behavior and equality in health care distribution. SDG-3 aims to ensure healthy lives and promote well-being for all at all ages [6]. Therefore, mother and newborn SSC becomes an essential practice to improve the health of the newborn [7]. It is against this backdrop that in the baby friendly hospital initiative (BFHI), there is a recommendation that newborns be placed in mother and newborn SSC for at least 1 h [8].

Generally, mother and newborn SSC helps in the newborn maintenance of blood glucose levels, temperature regulation, and metabolic adaptation. At birth, the newborn has a reduced capacity to generate heat, which results to a decline in body temperature. Hence, the maintenance of body temperature is paramount to the newborn at delivery. During mother and newborn SSC, there is a transfer of heat from the mother to her child, wherewith the mother’s body temperature activates the child’s sensory nerves, which in turn results in the child’s relaxation, reduces the tone of the sympathetic nerves, dilation of skin vessels and increase in its temperature [9]. Albeit, hypothermia during the newborn period has remained a factor of morbidity and mortality in resource-constrained settings [10]. High prevalence of hypothermia has been reported in countries with the highest rate of newborn mortality, where hypothermia is increasingly gaining attention as a major intervention for newborn survival [11].

Besides the provision of several benefits to the newborn, mother and newborn SSC has also been linked with many benefits for mothers. For example, the secretion of oxytocin in mothers who practice SSC strengthens uterine contractions, which in turn aids the placenta to separate and the duration of the third stage of labor is shortened [12]. The third stage of labor entails separation and expulsion of the placenta and begins immediately after childbirth [13]. In many health facilities, proper management of the third stage of labor is a common practice to quicken the process, in which synthetic oxytocin, which makes the uterus strongly contract is administered. In a spontaneous, uncomplicated birth, it is reasonable to plan a physiological or natural third stage by utilizing the mother’s own oxytocin [14]. As a simple, cost-effective and appropriate method, mother and newborn SSC is recommended to improve post-delivery care and potentially save the lives of mothers and newborns in particular [15].

Though WHO has recommended mother and newborn SSC, the separation of mothers and newborns is common in many health facilities whereby newborns are often placed under warmers or in cots [16]. In several SSA countries, mother and newborn SSC is rarely practiced [17], because mothers do not view SSC as important for keeping the baby warm after birth since newborns are usually taking away from their mothers immediately after birth, also due to prevailing cultural beliefs and practices that discourage mother and newborn SSC [18–20]. Moreover, health providers’ factors such as lack of skilled personnel and time constraint, lack of awareness of the practice and its benefit have accounted for low coverage of mother and newborn SSC [21]. Very low rates were reported in Tanzania (less than 1%) [22], Uganda and Mali (2% each) [23, 24], Nigeria (10%) [25], Ghana, (10%) [20], and in Ethiopia (9% and 13%) [26, 27]. To the best of our knowledge, there is no study in The Gambia that has reported the prevalence and determinants of mother and newborn SSC. We undertook this study to explore the prevalence and factors associated with mother and newborn SSC in The Gambia.

## Methods

### Data source

We used secondary data from The Gambia MICS—2018. Data from 9205 women aged 15-49 years who gave birth in the last 5 years was extracted for analysis. The data was based on the questionnaire for individual women administered in each household to all women of reproductive age (15-49 years). The United Nations Children’s Fund (UNICEF) developed the MICS program in the 1990s as an international household survey program to support countries in the collection of internationally comparable data on a wide range of indicators. MICS measure key indicators that allow countries to generate data for use in policies and programs, and to monitor progress toward the Sustainable Development Goals (SDGs), as well as the National Development Plan (NDP) of The Gambia and other internationally agreed upon commitments. In The Gambia, five Multiple Indicator Cluster Surveys have been conducted: 1996, 2000, 2006, 2010 and 2018. MICS6 contributed to the improvement of data and monitoring systems in The Gambia and strengthened technical expertise in the design, implementation, and analysis of such systems. The data can be downloaded online; https://mics.unicef.org/surveys.

### Sampling design

The sample for The Gambia MICS 2018 was designed to provide estimates for a large number of indicators on the situation of children and women at the national level, for urban and rural areas, and for the eight local government areas: Banjul, Kanifing, Brikama, Mansakonko, Kerewan, Kuntaur, Janjanbureh, and Basse. The urban and rural areas within each local government area were identified as the main sampling strata and the sample of households was selected in two stages. Within each stratum, a specified number of census enumeration areas were selected systematically with probability proportional to size. After a household listing was carried out within the selected enumeration areas, a systematic sample of 20 households was drawn in each sample enumeration area. All enumeration areas selected were visited during the fieldwork period. The procedures and standard guidelines developed under the global MICS program were adapted to The Gambia MICS 2018 final questionnaires and used throughout the survey [28].

### Variables selection and measurement

#### Outcome variable

Mother and newborn SSC was examined. The outcome was measured dichotomously as “1” if yes (had mother and newborn SSC) and “0” if otherwise (did not have mother and newborn SSC). Based on the definition of mother and newborn SSC, the outcome variable was measured in MICS-2018 using validated questionnaire for women in reproductive age (15-49 years).

#### Explanatory variables

Independent variables: household wealth quintile: poorest, poorer, middle, richer, and richest, was computed by MICS in a conventional approach from population-based data using a set of household assets [29, 30]; child sex: male vs. female; ethnicity: Mandinka, Wollof, Fula, Jola, Sarahule, other groups and non-Gambians; health insurance coverage: insured vs. not insured; educational level: pre-primary or none, primary, secondary+, functionality difficulty: has functional difficulty vs. has no functional difficulty; total children ever born: 1-2, 3-4, 5+; marital status: currently married/in union, formerly married/in union, never married/in union; residential status: urban vs. rural; age at first marriage/union: < 18 years, 18-20 years, 20+ years; estimation of overall happiness: very happy, somewhat happy, neither happy nor unhappy, somewhat unhappy, very unhappy; duration in residence: internal immigrant (lived in community < 5 years) vs. native (lived in community for 5+ years); maternal age (years): 15-19, 20-24, 25-29, 30-34, 35-39, 40-44, 45-49; frequency of listening to radio or watching TV: not at all, less than once a week, at least once a week, almost every day; received ANC: yes vs. no; ANC visit initiation: early booking (within 1st trimester) vs. late booking (after 1st trimester); place of delivery: home vs. health facility; birth weight: low birthweight (< 2.5 kg) vs. normal weight (at least 2.5 kg); local government area: Banjul, Kanifing, Brikama, Mansakonko, Kerewan, Kuntaur, Janjanbureh, Basse. Some of these were selected as used by previous authors [31].

### Ethical consideration

In this study, we utilized population-based dataset available in online with all identifier information removed. The survey protocol was approved by The Gambia Government and Medical Research Council Scientific Coordinating Committee (SCC) in March 2017. During the survey, verbal consent was obtained for each respondent participating and, for children age 15-17 years individually interviewed, adult consent was obtained in advance of the child’s assent. All respondents were informed of the voluntary nature of participation and the confidentiality and anonymity of information.

### Statistical analysis

Stata survey (‘svy’) module was used to adjust for stratification, clustering and sampling weights to compute the estimates of mother and newborn SSC. The collinearity testing approach adopted the correlation analysis to detect interdependence between variables. A cut-off of 0.7 was used to examine the multicollinearity known to cause major concerns [32]. No variable from the correlation matrix was removed in the model due to lack of multicollinearity. Percentages and chi-square test were used for analyses. All significant variables from chi-square test were included in the multivariable binary logistic regression model to calculate the adjusted odds ratios (with corresponding 95% CI) of the factors associated with mother and newborn SSC. In addition, we obtained the marginal predictive effects of the determinants of mother and newborn SSC [33]. Statistical significance was determined at *p* < 0.05. Data analysis was conducted using Stata Version 14 (StataCorp., College Station, TX, USA).

## Results

**Fig. 1:**
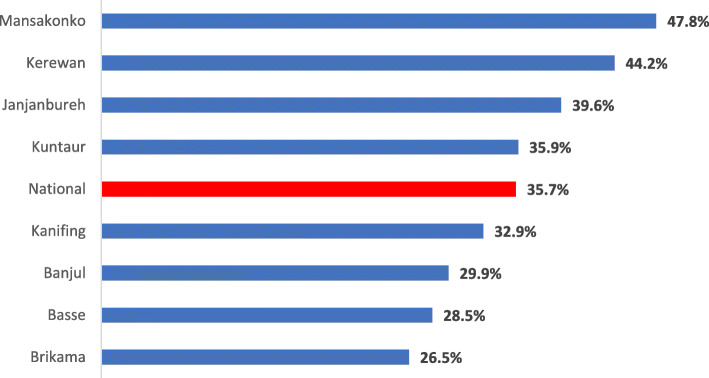
Local government areas discordance of mother and newborn skin-to-skin contact in The Gambia

The results of this study showed that the national prevalence of mother and newborn SSC was 35.7%. However, across the local government areas, Mansakonko (47.8%) and Kerewan (44.2%) had the highest prevalence of SSC in The Gambia. Conversely, Brikama (26.5%), Basse (28.5%), and Banjul (29.9%) had the least prevalence (Fig. 1).

Based on the results from Table 1, the percentage of mother and newborn SSC was about 38.6% for poorest household wealth quintile, 41.5% in Wollof ethnic group, approximately 38.9% in rural area, 38.9%, 40.3%, and 39.7% among those who booked early, gave birth at health facility and had normal weight baby respectively. See the table below for the details.

**Table 1:** Distribution of early mother and child skin-to-skin contact in The Gambia by selected socio-demographic and reproductive variables (*n* = 9205)

Variable	Number of respondents (%)	Mother and newborn skin-to-skin contact %		***P*** value (chi-square test)
		Yes	No	
**Sociodemographic profile**				
**Household wealth quintile (*n*** **= 9205)**				0.003*
Poorest	2936 (31.9)	38.6	61.4	
Second	2020 (21.9)	37.7	62.3	
Middle	1656 (18.0)	31.8	68.2	
Fourth	1335 (14.5)	32.0	68.0	
Richest	1258 (13.7)	32.0	68.0	
**Ethnicity (*n*** **= 9205)**				< 0.001*
Mandinka	2598 (28.2)	33.9	66.1	
Wollof	1503 (16.3)	41.5	58.5	
Fula	2317 (25.2)	37.9	62.1	
Jola	486 (5.3)	25.8	74.2	
Sarahule	952 (10.3)	33.6	66.4	
Other groups	649 (7.1)	34.6	65.4	
Non-Gambia	700 (7.6)	29.7	70.3	
**Residential area (9205)**				< 0.001*
Urban	4023 (43.7)	30.2	69.8	
Rural	5182 (56.3)	38.9	61.1	
**Frequency of watching TV (*n*** **= 9202)**^**β**^				0.009*
Not at all	3890 (42.3)	37.6	62.4	
Less than once a week	961 (10.4)	35.5	64.5	
At least once a week	1146 (12.5)	38.3	61.7	
Almost everyday	3205 (34.8)	31.9	68.1	
**Local government area (*n*** **= 9205)**				< 0.001*
Banjul	604 (6.6)	29.9	70.1	
Kanifing	1086 (11.8)	32.9	67.1	
Brikama	1297 (14.1)	26.5	73.5	
Mansakonko	973 (10.6)	47.8	52.2	
Kerewan	1072 (11.6)	44.2	55.8	
Kuntaur	1284 (13.9)	35.9	64.1	
Janjanbureh	1217 (13.2)	39.6	60.4	
Basse	1672 (18.2)	28.5	71.5	
**Reproductive profile**				
**ANC visit initiation (*n*** **= 3759)**^**β**^				< 0.001*
Early booking	1907 (50.7)	38.9	61.1	
Late booking	1852 (49.3)	32.4	67.6	
**Place of delivery (*n*** **= 3791)**^**β**^				< 0.001*
Home	831 (21.9)	19.4	80.6	
Health facility	2960 (78.1)	40.3	59.7	
**Birth weight (*n*** **= 3016)**^**β**^				0.003*
Low birthweight	310 (10.3)	31.0	69.0	
Normal	2706 (89.7)	39.7	60.3	

*Significant at *p* < 0.05; other factors examined but not statistically significant include child sex, health insurance coverage, functional difficulty, educational attainment, marital status, age at first marriage/union, estimation of overall happiness, duration in residence, maternal age, frequency of listening to radio, received ANC, total children ever born

^*β*^Did not sum up to the total sample (*n* = 9205) due to missing data among those who reported on the frequency of watching TV, timing to ANC visit initiation, place of delivery, and birth weight

Based on the results, there was 38% increase in the odds of rural women to report mother and newborn SSC, compared with the urban women after adjusting for other covariates (OR = 1.38; 95% CI: 1.06, 1.79). Mother and newborn SSC was significantly higher in Mansakonko local government area, when compared with Banjul local government area (OR = 1.94; 95% CI: 1.17, 3.19). Women who delivered at health facility were 3.35 times as likely to have mother and newborn SSC, compared with women who delivered at home after adjusting for other covariates (OR = 3.35; 95% CI: 2.37, 4.75). Conversely, women who initiated ANC after the first trimester had 21% reduction in the odds of mother and newborn SSC, compared with women who initiated ANC within the first trimester after adjusting for other covariates (OR = 0.79; 95% CI: 0.68, 0.93). Women who had normal weight (at least 2.5 kg) children were 1.37 times as likely to have mother and newborn SSC, compared with low birthweight (< 2.5 kg) children after adjusting for other covariates (OR = 1.37; 95% CI: 1.05, 1.78). See Table 2 for the details.

**Table 2:** Multivariable logistic regression of the determinants of mother and newborn skin-to-skin contact in The Gambia

Variable	Adjusted odds ratio	95%CI	***P***
**Sociodemographic profile**			
**Residential area**			
Urban	1.00		
Rural	1.38	1.06-1.79	0.017*
**Local government area**			
Banjul	1.00		
Kanifing	1.16	0.78-1.74	0.456
Brikama	0.89	0.58-1.36	0.586
Mansakonko	1.94	1.17-3.19	0.010*
Kerewan	1.23	0.76-2.00	0.392
Kuntaur	1.08	0.65-1.77	0.775
Janjanbureh	1.26	0.77-2.04	0.357
Basse	0.80	0.49-1.31	0.383
**Reproductive profile**			
**ANC visit initiation**			
Early booking	1.00		
Late booking	0.79	0.68-0.93	0.003*
**Place of delivery**			
Home	1.00		
Health facility	3.35	2.37-4.75	< 0.001*
**Birth weight**			
Low birthweight	1.00		
Normal	1.37	1.05-1.78	0.019*

*Significant at *p* < 0.05; model was adjusted for; household wealth quintile, ethnicity, and frequency of watching TV

In Table 3, marginal effect analysis was used to decipher the effects of the factors associated with mother and newborn SSC in The Gambia. It is clear, from the predictive marginal effects results, assuming the distribution of all factors remained the same among women, but every woman lived in rural residence or Mansakonko local government area, we would expect 42.0% and 52.0% mother and newborn SSC in The Gambia respectively. If instead the distribution of other maternal and child factors were as observed and other covariates remained the same among women, but all women had early ANC booking initiation, delivered at health facility or had normal weight baby, we would expect about 41.0% and 40.0% mother and newborn SSC in The Gambia respectively. In Table 3, we practically obtained the predictive marginal effects of the factors associated with mother and newborn SSC in The Gambia respectively. See Table 3 for the details.

**Table 3:** Marginal predictive model of the factors associated with mother and newborn skin-to-skin contact in The Gambia

Variable	Marginal effects	95% CI	***P***
**Sociodemographic profile**			
**Residential area**			
Urban	0.35	0.31-0.38	< 0.001*
Rural	0.42	0.39-0.45	< 0.001*
**Local government area**			
Banjul	0.36	0.27-0.45	< 0.001*
Kanifing	0.40	0.33-0.47	< 0.001*
Brikama	0.34	0.28-0.40	< 0.001*
Mansakonko	0.52	0.46-0.58	< 0.001*
Kerewan	0.41	0.36-0.46	< 0.001*
Kuntaur	0.38	0.33-0.43	< 0.001*
Janjanbureh	0.42	0.36-0.47	< 0.001*
Basse	0.32	0.27-0.36	< 0.001*
**Reproductive profile**			
**ANC visit initiation**			
Early booking	0.41	0.39-0.44	< 0.001*
Late booking	0.36	0.34-0.39	< 0.001*
**Place of delivery**			
Home	0.18	0.13-0.22	< 0.001*
Health facility	0.41	0.39-0.42	< 0.001*
**Birthweight**			
Low birthweight	0.33	0.27-0.38	< 0.001*
Normal	0.40	0.38-0.41	< 0.001*

*All factors were significant given *p* < 0.001; model was adjusted for; household wealth quintile, ethnicity and frequency of watching TV

## Discussion

The study has become the foremost to report the prevalence of mother and newborn SSC in The Gambia. Here, only about one-thirds of women reported mother and newborn SSC. This result is higher than the SSC prevalence of previous studies in Tanzania and Ethiopia. Less than 1% of Tanzanian women reported early SSC [22], and the proportion of women in Ethiopia who had early SSC after childbirth was 9.2% and 28.1% [26, 31]. However, the finding was low as compared to studies in high-income countries in which SSC practice rate was up to 99.0% [34]. The possible reasons for the low prevalence of mother and newborn SSC immediately after childbirth could be due to lack of enlightenment or awareness about its importance and such health information can be obtained through mass media [35]. The strain in delivery, social distress, and family burden can be a major concern for women [36], and prevent uptake of SSC. Furthermore, spousal support could in no small measure enhance the uptake of SSC [37]. For example, in a previous study, husbands whose wives utilized skilled delivery care provided instrumental, emotional, and informational support to their wives during delivery and understood that medical support was paramount in positive childbirth outcome [38]. Similarly, health care providers can play vital role to provide support for mothers during labor as well as aid post-delivery care uptake including the uptake of mother and newborn SSC [39]. In The Gambia, women have reported that emotional support from nurses was valued as the most helpful during childbirth [40].

According to the results of an experimental study [41], contact through the skin between women and their newborns had immediate advantages including that it helps to improve early initiation of breastfeeding, support the success of exclusive breastfeeding, maintains normal body temperature, maintains heart rate, respiratory rate, and blood pressure normal [16, 41, 42]. These benefits underscore the need to improve mother and newborn SSC. Among the determinants of SSC identified in this study, health facility-based delivery resulted in higher odds of mother and newborn SSC. This is consistent with the findings from previous study and quite expected because modern maternal health care delivery is hinged on skilled birth attendance [43]. This brings to limelight the advantages of institutional delivery including that trained health care professionals could provide specific care and attention to newborn babies with special needs in order to improve their survival chances and reducing the risk of maternal mortality. The reduction in the odds of home delivery could be blamed on lack of skilled birth attendants to assist the women in the practice or due to the fact that traditional birth attendant may lack the scientific information of SSC and its benefits.

Furthermore, women from rural area were found to have increased odds for mother and newborn SSC. This finding could be due to the fact that The Gambia is predominantly rural, as such, mother and newborn SSC could be higher in rural residence because it is possible that more health facilities are located in rural areas. In addition, women who had children with normal birth weight accounted for higher odds of mother and newborn SSC. This is consistent with the results of a previous study wherewith appropriate birth weight was associated with higher prevalence of skin to skin contact [44]. In this study, we found that proper fetal care during pregnancy resulting in optimal birthweight, was associated with improved mother and newborn SSC. These findings reinforce the hypothesis of strong influence of ANC practices and baby’s conditions at birth in enhancing SSC between mother and newborn.

Conversely, women who reported late booking in ANC initiation had reduction in the odds of mother and newborn SSC. In a previous study, increased number of ANC visits was associated with higher SSC practice during the postpartum stay. Interestingly, early ANC initiation has been reported to be associated with higher number of ANC visits [45]. This explains why women with late ANC booking had reduction in the odds of mother and newborn SSC. When mothers attend more ANC visits or keep ANC appointments regularly, their awareness will increase thereby having good knowledge on the purpose of SSC and adherence to care for them and the newborn become more effective, also, this will result to large compliance, and finally leads mothers to provide SSC to their newborns. In addition, geographical location was significantly associated with mother and newborn SSC. This is consistent with the findings from a previous study [46]. The change in the odds of mother and newborn SSC in certain geographical location such as local government areas could be due to differences in availability, accessibility, and efficiency of health care delivery in such location. Again, previous maternal and newborn health care intervention in certain locations and exposure to certain behavior change communication could also explain or enhance the uptake of related health care practices.

### Strengths and limitations

This study utilized large sample size, giving a national representative study sample. Such population-level findings for newborn care practices provide valuable knowledge for a health area where such data are practically lacking. Although the data are representative of the study area, it is likely that frequencies of newborn care behaviors vary between different geographies in The Gambia. For example, questions about delivery and newborn care practices could be restricted to women who had delivered within few years preceding the interview to maximize recall accuracy. The cross sectional nature of the present study prevents any causal inferences. The use of secondary data limited essential variables such as demand-side factors needed to understand in-depth newborn care practices. Although this study included all women who have given birth in the last 5 years to the survey, however, the large low response rate in maternal profile variables could have biased our estimates.

## Conclusion

The prevalence of mother and newborn SSC was low. In addition, geographical residence, birth weight, urban-rural residential status, place of delivery, and timing to ANC initiation were associated with mother and newborn SSC. These suggest the importance of ANC as an appropriate time to inform pregnant women about mother and newborn SSC. However, the need to adopt measures that prioritize mother and newborn SSC in the delivery room is emphasized. Women should know about the importance of these practices and be encouraged to practice it. Also, there is a need to prioritize training of health providers on the implementation of essential newborn care including SSC. Community engagement is also needed to ensure that all women and their families understand the benefits of SSC.

## Data Availability

Unique identifiers such as location and names collected during interviews were removed from datasets to ensure confedintiality. These anonymized data files are made available and can be freely downloaded for legitimate research purposes on; https://mics.unicef.org/surveys.

## Abbreviations

ANC: Antenatal care
BFHI: Baby friendly hospital initiative
CI: Confidence interval
MICS: Multiple indicator cluster survey
NDP: National development plan
OR: Odds ratio
SCC: Scientific coordinating committee
SDG: Sustainable development goal
SSA: Sub-Saharan Africa
SSC: Skin-to-skin contact
UNICEF: United Nations Children’s Fund
WHO: World Health Organization
